# Relationship between water hardness, pH, and organic acid requirement for effective water acidification in swine operations

**DOI:** 10.1093/tas/txaf164

**Published:** 2025-12-14

**Authors:** Maxwell L Corso, Julian Arroyave, Jason C Woodworth, Mike D Tokach, Robert D Goodband, Joel M DeRouchey, Jordan T Gebhardt, Ganga M Hettiarachchi

**Affiliations:** Department of Animal Sciences and Industry, College of Agriculture, Kansas State University, Manhattan, KS 66506-0201, United States; Department of Animal Sciences and Industry, College of Agriculture, Kansas State University, Manhattan, KS 66506-0201, United States; Department of Animal Sciences and Industry, College of Agriculture, Kansas State University, Manhattan, KS 66506-0201, United States; Department of Animal Sciences and Industry, College of Agriculture, Kansas State University, Manhattan, KS 66506-0201, United States; Department of Animal Sciences and Industry, College of Agriculture, Kansas State University, Manhattan, KS 66506-0201, United States; Department of Animal Sciences and Industry, College of Agriculture, Kansas State University, Manhattan, KS 66506-0201, United States; Department of Diagnostic Medicine/Pathobiology, College of Veterinary Medicine, Kansas State University, Manhattan, KS 66506-0201, United States; Department of Agronomy, College of Agriculture, Kansas State University, Manhattan, KS 66506-0201, United States

**Keywords:** acid, calcium, citric acid, magnesium, water hardness, water pH

## Abstract

A total of 45 water samples from swine production sites across six states were used to determine the amount of CitraSol (Northwest Livestock Distribution, Medina, MN), a citric acid product, required to reduce water pH to a common end point. Water samples were analyzed for pH, Ca, Mg, and hardness. Total hardness was calculated using Ca and Mg concentrations and expressed as mg of CaCO_3_/L. Water hardness ranged from 142 to 1,181 mg CaCO_3_/L with an average of 441.2 mg CaCO_3_/L. Initial pH ranged from 7.42 to 8.47 with an average of 7.91. In triplicate, CitraSol was added to 10 mL samples of each water source to reach a stable pH of 5.0 and 4.0 ± 0.05. An inverse relationship between water hardness and initial pH was observed (quadratic, *P = *0.002; R^2^ = 0.22). The amount of CitraSol required to reach a stable pH of 4.0 increased (quadratic, *P <*0.001) as hardness, Ca, and Mg increased (R^2 ^= 0.30, 0.27, 0.28, respectively). Surprisingly, high initial pH water required less (quadratic, *P <*0.001; R^2^ = 0.31) CitraSol to reach a pH of 4.0. We hypothesize this was partially due to the reduction in the amount of free Ca ions as the water becomes more alkaline in nature. A sub sample of the water samples was titrated using Activate WD Max (Novus International, Chesterfield, MO) to determine if the amount of acid required to reduce water pH to the same common end point was acid specific. A direct relationship between the amount of CitraSol and Activate WD Max (linear, *P <*0.001; R^2^ = 0.87) to reach a pH of 4.0 was observed, suggesting that data from one acid may allow prediction of the quantity required of another acid to reach the same target pH. Similarly, titrating to a pH of 5.0 can predict the amount of acid required to reach a pH of 4.0 (linear, *P <*0.001; R^2^ = 0.99). In conclusion, pH, Ca, Mg, and hardness cannot fully predict the amount of acid required to reach a stable water pH of 4.0. However, relationships were observed that can partially explain the variation in the amount of acid required. This data suggests that acid titrations of individual water sources should be completed to determine the amount of acid required to reach a final pH of 4.0.

## Teaser

Individual water sources for swine require different levels of acidifiers to reach a final targeted water pH.

## Introduction

Water is a fundamental nutrient required for all living organisms and is essential for normal metabolic functions including body temperature regulation, waste excretion, and is necessary to maximize feed consumption (NRC 2012). Although necessary for survival, water research has received little attention, especially in livestock production (Patience 2012). Research involving water has primarily focused on evaluating the impact of water quality on growth performance (Anderson et al. 1994; Lozinski et al. 2022) and quantifying water utilization under different environmental conditions or management scenarios (Nannoni et al. 2013; Schale et al. 2023). Water quality can be evaluated using three broad criteria including: physical, chemical, and microbiological characteristics, with most of the attention placed on chemical and microbiological criteria. When evaluating chemical criteria, the focus is mainly on the concentration of macro and micro minerals, pH, nitrate, sulfates, heavy metals, etc (Saalidong et al. 2022). Water hardness is defined as the concentration of divalent cations, primarily Calcium (Ca^2+^) and Magnesium (Mg^2+^). Hard water contains high concentrations of these minerals (Büker et al. 2021), which can influence several chemical and biological processes. From a chemical perspective, hard water has greater buffering capacity, making it more resistant to pH changes (Stumm and Morgan 1996). However, elevated hardness can also affect solubility and bioavailability of nutrients and minerals in animal drinking water, as Ca^2+^ and Mg^2+^ ions may form precipitates or complexes with other compounds (Boyd 2015).

The chemical composition of the water can vary based on well location, design, and depth (Patience 2012). Although not demonstrated to be a health concern to the pig, elevated levels of hardness, or the additive concentrations of Ca and Mg salts in the water source can lead to scale build up in water delivery and treatment systems (Patience 2013). The accumulation of mineral deposits and biofilm inside water lines can impair the function of water filters and nipple drinkers, thereby reducing water flow rate (Patience 2013). Water acidification has been proposed as a practical strategy to clean water lines, limit microbial growth, and improve animal performance and health. Studies have reported beneficial effects of acidified water on growth performance and gut health in pigs (Gottlob et al. 2005; Xu et al. 2022) and broilers (Parker et al. 2007).

The use of acidifiers led to a reduction in pH because organic acids dissolve in water and dissociate to release hydrogen ions (H^+^), thereby increasing the proton concentration in solution and lowering the pH (Stumm and Morgan 1996). The effectiveness of an acidifier depends on its acid dissociation constant (pKₐ) and the buffering capacity of the solution (Price et al. 2020). When acids are added into water containing bicarbonate (${\text{HCO}}_{3}^{-}$) and carbonate (${\text{CO}}_{3}^{2-}$) ions, these buffer species consume H^+^ limiting the reduction in pH (Essington 2015). In addition, water hardness may also contribute to the effectiveness of an acidifier to change the solution pH. Elevated concentrations of Ca and Mg may interact with other water components such as chlorides, carbonates, and sulfates forming precipitates as the pH changes, reducing the number of ions that can interact with the available H^+^ for lowering pH (Essington 2015). Thus, acidifier efficacy is influenced not only by the strength of the acid but also by the total mineral content.

There are many commercially available water acidifiers including organic, inorganic, and acid-blend products. Citric acid is a commonly used organic acid because it is relatively inexpensive and readily accessible. The commercial acidifier, Activate WD Max (Novus International, Chesterfield, MO), is an organic acid blend that can be used as an alternative to citric acid. We hypothesized that certain chemical characteristics of water such as Ca and Mg concentrations might negatively influence the ability of acidifiers to reduce water pH. Therefore, the objective of this study was to determine if different water quality characteristics associated with different water sources influence the amount of acidifier added to reach a stable pH endpoint of 5.0 and 4.0.

## Materials and methods

### General

A total of 45 water samples were collected from commercial swine production facilities located in Iowa, Kansas, Minnesota, Missouri, Ohio, and Tennessee. Samples represented both municipal and well water and were obtained directly from drinkers using 500-mL wide-mouth round bottles with white polypropylene caps (Fisher Scientific, Kansas City, KS). Following collection, all samples were shipped to Kansas State University and stored at 4°C until chemical analyses were conducted.

### Chemical analysis

A 25 mL subsample of each water sample was sent to the Kansas State University Soil Testing Laboratory, Manhattan, KS, for analysis. Initially, samples were filtered through a 0.45 µm filter, then pH was determined using a Seven Direct SD50 pH/Ion meter (Mettler Toledo, Columbus, OH) which was calibrated with pH 4 and 7 buffer solutions (Fisher Chemica, Pittsburgh, PA). Calcium and Mg concentrations were analyzed using inductively plasma-optical emission spectrometry (ICP-OES) to determine Ca, and Mg following the EPA SW-846 Method 6010D (Table 1). Analyzed Ca and Mg concentrations were then used to calculate total hardness expressed in calcium carbonate equivalence using the equation described by Boyd (2015): Total Hardness, mg CaCO_3_/L = (Ca, mg/L × 2.479) + (Mg, mg/L × 4.118).

**Table 1.** Range of analyzed water characteristics^a^.

Variable	Minimum	Average	Maximum
**Sample pH**	7.42	7.91	8.47
**Calcium, mg/L**	34	110	300
**Magnesium, mg/L**	9	40	105
**Total hardness, mg CaCO_3_/L^b^**	142	441	1,181

a A total of 45 swine production-site water samples across six states were collected and analyzed.

b Total hardness, mg CaCO_3_/L = (Ca, mg/L × 2.479) + (Mg, mg/L × 4.118).

To estimate the amount of citric acid (CitraSol; Northwest Livestock Distribution, Medina, MN) required to reduce the water pH to target values of 5.0 and 4.0 ± 0.05, two 10-mL subsamples from each bottle were titrated with increasing levels of the organic acid until the desired pH was achieved. To analyze water pH during the acid titration process, a Mettler Toledo Seven Compact SS20 pH/Ion meter (Mettler Toledo, Columbus, OH) was used. During each titration, the water samples were placed in a 100 mL beaker containing a metal stir bar and the beaker was located on a stir plate to continuously homogenize the water sample. The amount of CitraSol required to reach each pH was recorded for each replicate and the average was calculated for each sample for each pH level.

To determine the amount of a second acidifier needed to lower water pH to target values of 4.0 or 5.0, a subset of 13 water samples was used to test Activate WD Max (Novus International, Chesterfield, MO), an organic acid blend containing lactic acid, phosphoric acid, and methionine hydroxy analogue. Titration was performed using the same procedure as for CitraSol to compare the quantities of each acidifier required to achieve the desired pH.

### Statistical analysis

Data were analyzed using R Studio (Version 4.3.0, 2023-03-01; R Foundation for Statistical Computing, Vienna, Austria). Linear and quadratic relationships between starting pH, hardness, Ca and Mg concentrations and the amount of CitraSol required to reach the target pH were estimated using the lm function. For each pair of variables, two models (first- and second-order) were developed. Model selection was based on both the coefficient of determination (R^2^) and the significance of the linear and quadratic terms. When both models were statistically significant, the higher-order model (quadratic) was chosen to best describe the relationship. Graphical representations of the selected models were generated using the ggplot function from the ggplot2 library. Following the same methodology, the relationship between the amounts of CitraSol and Activate WD Max required to reach the same target pH was also analyzed. All results were considered significant at *P* ≤ 0.05.

## Results and discussion

Patience (2012) described three broad criteria that are used when evaluating water quality. These include physical, microbiological, and chemical properties. Physical criteria include qualities, which are believed to be of minimal consideration in pig production, including color, turbidity, and odor. Most attention is placed on the microbiological properties including microbial growth and contamination; along with chemical properties including concentrations of Ca, Mg, sulfates, nitrates, and other minerals.

When evaluating the chemical properties, total hardness can be used to characterize water quality. Hardness is the measure of the sum of divalent cations including Ca and Mg in the water (Nyachoti et al. 2022), and is expressed as calcium carbonate (CaCO_3_) equivalence (Essington 2015). Hardness, for water supply purposes, is often classified into four categories; soft (<50 mg/L), moderately hard (50 to 150 mg/L), hard (150 to 300 mg/L), and very hard (>300 mg/L; Boyd 2015). Geographic location and water source (municipal vs well water) are critical variables that directly affect water hardness. Across the 45 water samples collected from six states, hardness ranged from 142 to 1,181 mg CaCO_3_/L, with an average of 441.2 mg CaCO_3_/L. Initial pH values ranged from 7.42 to 8.47, averaging 7.9 (Table 1). Based on the Boyd (2015) classification, all samples were considered as very hard and alkaline according to the pH.

**Fig. 1.**
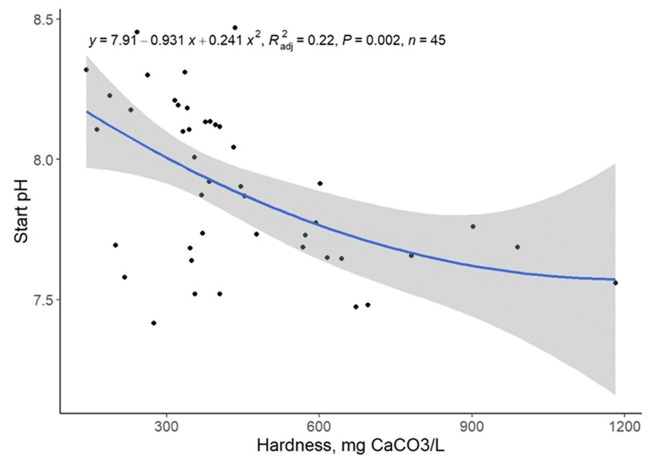
Relationship between starting water pH and hardness. Black dots represent independent observations, the blue line depicts the predicted relationship between variables, and the gray shaded area indicates the 95% confidence interval.

Low pH values indicate acidic conditions, which result from higher concentrations of hydrogen ions in the solution. In contrast, solutions with greater concentrations of Ca^2+^ and Mg^2+^ typically have higher alkalinity and buffering capacity, which resist decreases in pH by neutralizing hydrogen ions (Stumm and Morgan 1996). Consequently, it was logical to expect a positive linear relationship between water hardness and pH. However, opposite to expectations, an inverse relationship between sample hardness and initial pH was observed (quadratic, *P = *0.002; R^2^ = 0.22; Table 2, Fig. 1), where an increase in sample hardness resulted in a lower starting pH.

**Table 2.** Relationship between water characteristics and CitraSol^a^ (mL) required to reach a stable pH of 4.0^b^.

	Linear		Quadratic	
Relationship	*P*	R^b^	*P*	R^b^
**Start pH and hardness**	<0.001	0.22	0.002	0.22
**Acid required (mL) to reach pH of 4.0 relationship with**				
**Hardness**	0.001	0.19	<0.001	0.30
** Ca concentration**	0.002	0.19	<0.001	0.27
** Mg concentration**	0.002	0.18	<0.001	0.28
** Starting pH**	<0.001	0.31	<0.001	0.31
**Acid required (mL) to reach pH of 5.0**	<0.001	0.99	…	…

a CitraSol, Northwest Livestock Distribution, Medina, MN.

b A total of 45 swine production-site water samples across six states were collected and analyzed.

The total Ca^2+^ and Mg^2+^ in solution represents the sum of free ions and soluble complexes (Essington 2015). However, in hardness calculations, no distinction is made between these chemical forms. The relative proportion of each form may help explain the negative relationship between hardness and pH observed in the current study. When the Ca and Mg in the water are present in the form of chlorides or sulfates, such as CaCl^+^, ${\text{CaCl}}_{2}^{0}$, ${\text{CaSO}}_{4}^{0}$, ${\text{MgSO}}_{4}^{0}$, etc their participation in acid–base equilibria is limited because they are no longer available to interact with carbonate or hydroxide ions. Consequently, complexed species do not contribute to neutralization or buffering reactions that determine hydrogen ion activity and, therefore, exert minimal influence on water pH (Stumm and Morgan 1996; Essington 2015). These complexes, however, still contribute to total hardness. Thus, an increase in the proportion of complexed Ca and Mg may lead to greater hardness without a corresponding change in pH, as previously reported by (Büker et al. 2021).

Although differentiating between free and complexed forms of Ca and Mg is essential to explain the relationship between pH and hardness, the relative abundance of each chemical form was not estimated in the current study. Direct analysis of free ion species or soluble complexes of Ca and Mg is technically challenging, and analytical methods are not widely available. Consequently, the total dissolved concentration of each element is typically measured, and the distribution between free and complexed species is estimated using established chemical principles and equilibrium models (Essington 2015). Assuming that cations present within water (Ca^2+^, Mg^2+^, and others) form only one complex with each ligand (a molecule or ion capable of forming a coordination complex with a metal), a typical water source can contain over 100 soluble complexes and free species making a water source extremely complex (Essington 2015). For example, in alkaline water, bicarbonate is the major inorganic carbon species and preferentially reacts with hard cations, which are considered acids with low polarizability according to the hard and soft acid–base theory from Person (electron-pair acceptor), such as Ca^2+^ and Mg^2+^ (Pearson 1963, 1968), forming calcium and magnesium carbonate.

**Fig. 2.**
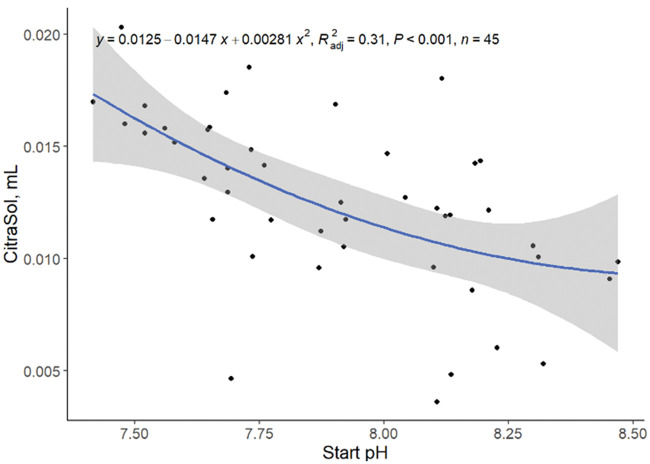
Relationship between mL of CtiraSol required to reach a pH of 4.0 and water starting pH. Black dots represent independent observations, the blue line depicts the predicted relationship between variables, and the gray shaded area indicates the 95% confidence interval.

High initial sample pH was associated with a reduction (quadratic, *P <*0.001; R^2^ = 0.31; Fig. 2) in the amount of CitraSol required to reach a pH of 4.0. This is likely because citric acid can effectively dissociate and release more protons when solution pH is higher. The pKa values of citric acid are: pKa1 = 3.13, pKa2 = 4.76, and pKa3 = 6.40. This means that at pH 3.13, only 50% of the first carboxylic group (out of all three groups) are dissociated and provide H^+^ to the solution. As pH increases, more of that first carboxylic group dissociates, giving H^+^ to the solution. Similarly at pH 4.76, only 50% of that second carboxylic group is dissociated. As pH increases, more of it becomes dissociated. The same goes with the third carboxylic group. This means that the higher the pH, the more dissociated the three carboxylic groups in citric acid are, releasing more H^+^ and helping to neutralize the pH and providing more negative sites are available for binding with cationic Ca and Mg.

Essington (2015), stated that close to 100% of the total K, Na, Cl, and NO_3_ in alkaline water occur as free species. However, only 89% percent of the total Ca and 88% of the total SO_4_ occur as free species; resulting in less Ca available to bind the added CitraSol. Conversely, Essington (2015) showed that free, uncomplexed species of Ca^2+^, Mg^2+^, K^+^, Na^+^, ${\text{SO}}_{4}^{2–}$, Cl^–^, and ${\text{NO}}_{3}^{–}$ dominate acidic water solutions. Additionally, acidic water contains approximately 100% of the total Ca, Mg, K, Na, Cl, and NO_3_ and 84% of the total SO_4,_ as the free species. Thus, more uncomplexed species present in lower pH water are available to bind to the added acidifier meaning that an increase in the amount of acidifier is required to lower pH in acidic water compared to alkaline water. Sample pH does not fully predict (R^2^ = 0.31; Fig. 2) the variation in the amount of CitraSol required to reduce water pH to 4.0. We speculate that it is possible that metal citrate precipitates may be forming in water with lower starting pH, resulting in greater amount of acid required to lower water pH (G. M. Hettiarachchi 2024, personal communication). A second explanation might be that some partially dissociated citric acid (ie, citric acid with only one or two dissociated carboxylic groups) can complex with free metal cations.

**Fig. 3.**
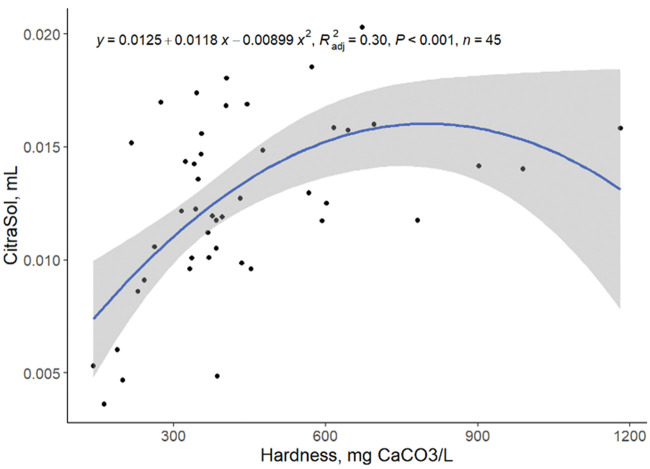
Relationship between mL of CitraSol required to reach a pH of 4.0 and water hardness. Black dots represent independent observations, the blue line depicts the predicted relationship between variables, and the gray shaded area indicates the 95% confidence interval.

**Fig. 4.**
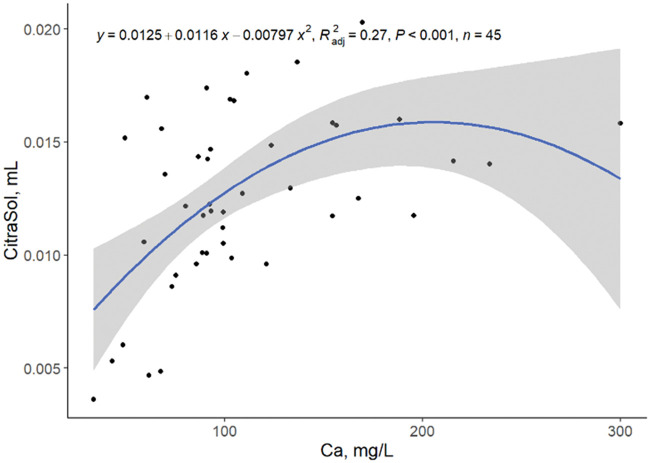
Relationship between mL of CitraSol required to reach a pH of 4.0 and water Ca concentration. Black dots represent independent observations, the blue line depicts the predicted relationship between variables, and the gray shaded area indicates the 95% confidence interval.

**Fig. 5.**
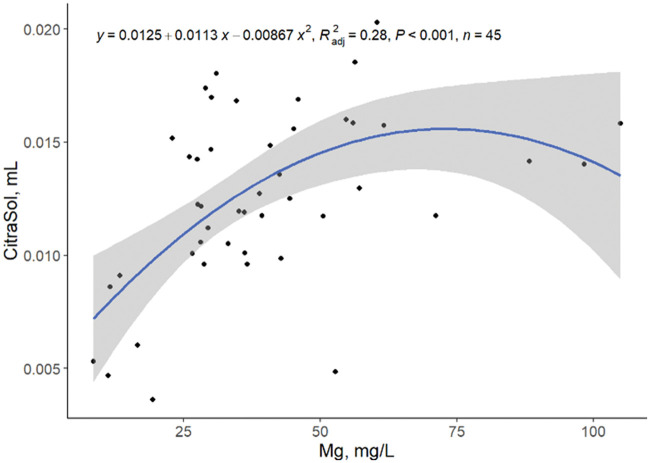
Relationship between mL of CitraSol required to reach a pH of 4.0 and water Mg concentration. Black dots represent independent observations, the blue line depicts the predicted relationship between variables, and the gray shaded area indicates the 95% confidence interval.

**Fig. 6.**
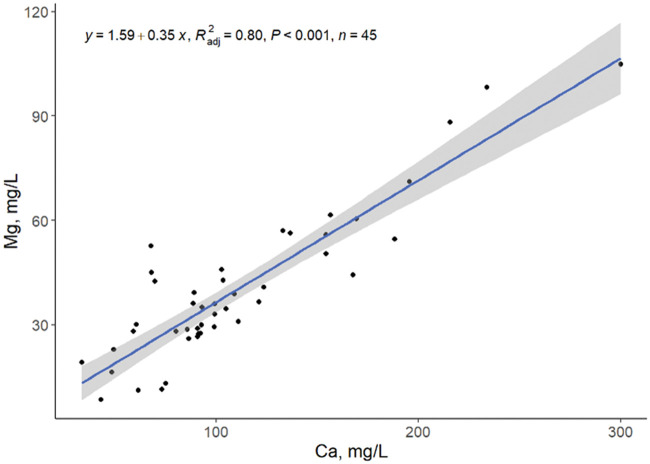
Relationship between water Mg and Ca concentrations. Black dots represent independent observations, the blue line depicts the predicted relationship between variables, and the gray shaded area indicates the 95% confidence interval.

A low correlation between the amount of CitraSol to obtain a stable pH of 4.0 and hardness, Ca, and Mg as single predictors was observed (Figures 3–5), leading us to conclude that there are other variables that affect the ability of an acidifier to reduce water pH. Although species availability can be estimated by water pH, element species distribution is environment and element specific (Essington 2015). The amount of CitraSol required to reach a sample pH of 4.0 increased (quadratic, *P* *<* 0.001) as hardness, Ca, and Mg increased (R^2^ = 0.30, 0.27, 0.28, respectively). This was as expected due to the acid binding characteristics of both Ca and Mg. A high correlation between Ca and Mg concentrations was observed (linear, *P <*0.001; R^2^ = 0.80; Fig. 6) concluding that Ca and Mg concentrations are related and an increase in the concentration of one of these minerals is often associated with an increase of the other.

**Fig. 7.**
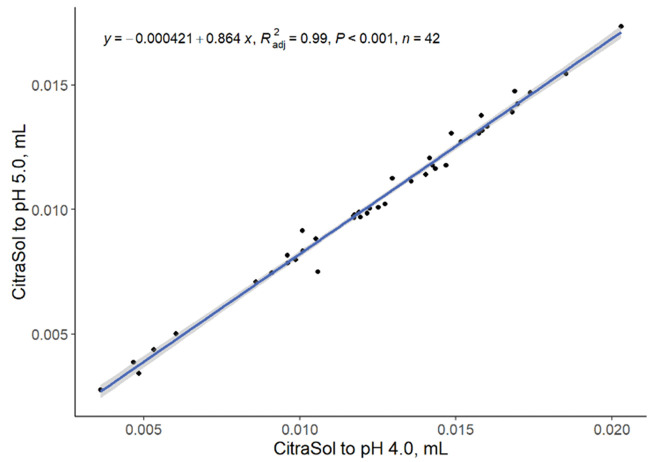
Relationship between quantity of CitraSol required to reach a pH of 5.0 and 4.0 in water samples. Black dots represent independent observations, the blue line depicts the predicted relationship between variables, and the gray shaded area indicates the 95% confidence interval.

Adding CitraSol to reach a target pH of 5.0 can predict (linear, *P < *0.001; R^2^ = 0.99; Fig. 7) the amount of acid required to reach a pH of 4.0. Therefore, the amount of CitraSol required to reach one pH endpoint can be used to predict the amount of CitraSol required to reach a lower pH endpoint.

**Fig. 8.**
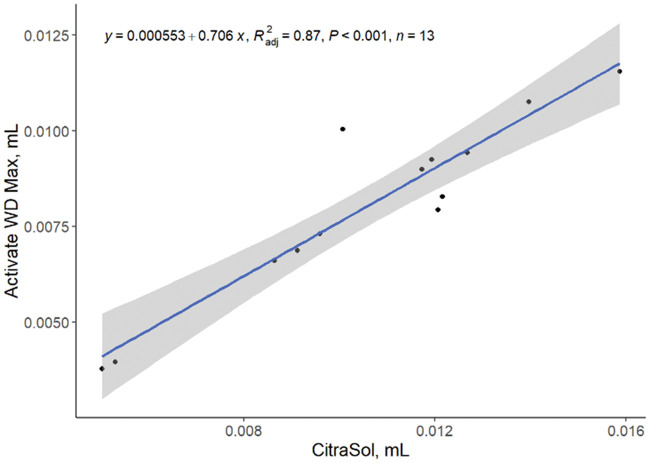
Relationship between amount of CitraSol and activate WD max required to each a pH of 4.0 in water samples. Black dots represent independent observations, the blue line depicts the predicted relationship between variables, and the gray shaded area indicates the 95% confidence interval.

When also using a second organic acidifier (Activate WD Max), it was determined that the amount of acid required to reduce water pH was specific to the acid used in the titration. A direct relationship between the amount of CitraSol and Activate WD Max, (linear, *P < *0.001; R^2^ = 0.87; Fig. 8) to reach a pH of 4.0 was observed, suggesting that the amount of these organic acidifiers required to reach a target pH can be used to predict the amount of a different organic acidifier needed to reach the same target pH endpoint.

The current results demonstrate that water hardness can influence the effectiveness of compounds such as acidifiers in achieving a target pH. Because mineral composition varied widely among the water samples collected in this experiment, it is evident that a single, universal protocol for acidifier use is not feasible. Instead, acid concentrations and application strategies should be evaluated on a farm-specific basis to ensure the desired pH is reached. Moreover, variations in acidifier protocols may affect not only pH control but also the efficacy of water-delivered medications and the growth of waterborne microorganisms. These findings highlight the need for further research to optimize water treatment strategies, particularly as water is increasingly used as a vehicle for nutrients and medications in swine production systems.

In conclusion, water pH, Ca, Mg, and hardness cannot fully predict the amount of CitraSol required to reach a stable pH of 4.0. Therefore, titration of specific organic acidifiers in unique water sources is necessary to determine the amount required to reach a final target water pH.

## Abbreviations:

ABC-4: acid binding capacity-4
Ca: calcium
CaCO_3_: calcium carbonate
Mg: magnesium
